# Metabolic reprogramming and metabolic sensors in KSHV-induced cancers and KSHV infection

**DOI:** 10.1186/s13578-021-00688-0

**Published:** 2021-09-27

**Authors:** Tingting Li, Shou-Jiang Gao

**Affiliations:** Cancer Virology Program, UPMC Hillman Cancer Center, Department of Microbiology and Molecular Genetics, University of Pittsburgh School of Medicine, Pittsburgh, PA 15213 USA

**Keywords:** Metabolic reprogramming, Metabolic sensors, Kaposi’s sarcoma-associated herpesvirus, KSHV, Kaposi’s sarcoma, Primary effusion lymphoma

## Abstract

Kaposi’s sarcoma-associated herpesvirus (KSHV) is an oncogenic gammaherpesvirus associated with several human cancers. KSHV infection and KSHV-induced anabolic cell proliferation and cellular transformation depend on reprogramming of cellular metabolic pathways, which provide the building blocks and energy for the growth of both the virus and the infected cells. Furthermore, KSHV dysregulates numerous metabolic sensors including mTOR, AMPK, CASTOR1 and sirtuins to maintain cellular energetic homeostasis during infection and in KSHV-induced cancers. In this review, we summarize the recent advances in the understanding of KSHV hijacking of metabolic pathways and sensors, providing insights into the molecular basis of KSHV infection and KSHV-induced oncogenesis. In addition, we highlight the critical metabolic targets and sensors for developing potential new therapies against KSHV infection and KSHV-induced cancers.

## Introduction

Cancer is recognized as a metabolic disease since a defining hallmark of cancer is uncontrolled proliferation that demands surplus bioenergetics and biosynthetic precursors [1]. To meet these demands, cancer cells rewire metabolic pathways by dysregulating tumor suppressors or oncogenes. Conversely, cancer cells produce oncometabolites that further activate oncogenic signaling pathways to support cell proliferation and survival [2].

According to the assessment of the International Agency for Research on Cancer, viral infection accounts for at least 11% of human cancer worldwide [3]. Oncogenic viruses induce tumorigenesis by chronically infecting host cells and consequently inducing genetic and persistent epigenetic alterations of the host cells resulting in the dysregulations of cellular oncogenic and tumor suppressor pathways. Kaposi’s sarcoma-associated herpesvirus (KSHV) is an oncogenic gammaherpesvirus associated with several human cancers [4]. KSHV persistent infection is required for cellular transformation and tumorigenesis [5]. Similar to cancer, successful viral infection depends on reprogramming cell metabolic pathways, which provide the building blocks and energy for the replication of the virus as well as the survival and proliferation of the infected cells [6]. Emerging evidence has shown that KSHV hijacks the cellular catabolic and anabolic pathways to support the survival and proliferation of infected cells. A recent review focuses on how KSHV viral genes regulate host cell signaling pathways that could potentially impact the metabolic pathways [7]. In this review, we comprehensively summarize the direct regulation of host cell metabolic pathways during different stages of KSHV infection and cellular transformation.

## Cancer metabolism

The recent resurgence of research in cancer metabolism has arisen as a result of advances in newly developed biomedical and biological tools, which expands our understanding of the underpinning mechanisms and functional consequences of altered metabolism in cancer. Cancer cells often show increased consumption of glucose, accompanied by a switch of energy metabolism from oxidative phosphorylation to aerobic glycolysis even in the presence of ambient oxygen, the so-called Warburg effect [8]. Although the aerobic glycolysis has low energy (ATP) yield, it is widely regarded as a way to effectively provide precursors and NADH for the synthesis of biologically relevant macromolecules [9]. Glutamine is a primary source of both carbon and nitrogen for de novo synthesis of diverse nitrogen-containing building blocks including nucleotides, fatty acids and nonessential amino acids (arginine, proline, asparagine) [2]. Proliferating cancer cells are highly addicted to glutamine leading to accelerated glutamine uptake and glutaminolysis [2, 9]. Hence, glutamine deprivation often leads to cancer cell death and decreased cell proliferation, which is countered by glutamine anaplerosis [9]. DNA and RNA synthesis is the basis of cancer cell proliferation, which demands rapid nucleotides synthesis driven by c-Myc through upregulation of nucleotide biosynthesis enzymes [9]. Acetyl-CoA, which is derived from glucose and glutamine, is the building block of fatty acids and cholesterol. With the rapid division of cancer cells, the membrane synthesis supported by de novo fatty acids synthesis is increased. Additionally, one-carbon metabolism is a universal metabolic process in eukaryotes and across organs, and is frequently enhanced in cancer cells to support the biosynthesis of nucleic acids, counter the stress of reactive oxygen species (ROS) and control the concentration of three amino acids: glycine, methionine, and serine [10, 11]. Furthermore, as the only supply of the methyl group, one-carbon metabolism is frequently upregulated in cancer to provide the methyl group required for DNA, RNA, and histone modifications, which supports tumor progression by dysregulating gene expression [12]. Although the reprogramming of metabolic activities in cancer is widely documented, most data are obtained in vitro. How and to what extent this contributes to tumorigenesis in vivo remains largely unclear. A breakthrough in techniques that allow better determination of the in vivo conditions is urgently required.

## KSHV and KSHV-associated human diseases

Kaposi’s sarcoma-associated herpesvirus (KSHV), discovered in 1994 by Chang and Moore [13], is one of the seven oncogenic viruses and the causative agent of Kaposi’s sarcoma (KS), primary effusion lymphoma (PEL), multicentric Castleman’s diseases (MCD) and KSHV-associated inflammatory cytokine syndrome (KICS) [4, 14]. The life cycle of KHSV comprises two phases, known as the latent and lytic phases. During latency, KSHV only expresses a few genes including LANA (ORF73), vCyclin (ORF72) and vFLIP (ORF71) together with 25 microRNAs (miRNAs) derived from a cluster of 12 precursor miRNAs (pre-miRNAs) named KSHV-miR-K12-1-12 (hereafter referred to as miR-K1-12) [5, 15]. Among them, LANA is essential for KSHV episome maintenance in host cells by mediating viral genome replication and tethering viral genome to chromosomes to ensure appropriate segregation during mitosis [16]. By contrast, during lytic replication, KSHV expresses cascades of lytic genes among which replication and transcriptional activator (RTA) encoded by ORF50 is essential and sufficient for initiating KSHV lytic replication [5].

KS tumors are spindle-shaped cells expressing vascular endothelial, lymphatic endothelial, precursor and mesenchymal markers [17]. Most KS and PEL tumors are latently infected by KSHV, suggesting that KSHV latent infection is critical for KSHV-induced tumorigenesis [5]. On the other hand, KSHV lytic replication is required for the spread and infection of new cells [18]. Numerous KSHV lytic genes target oncogenic and tumor suppressive pathways, mediate cell survival and cellular proliferation, and induce inflammation [19–23]. Hence, KSHV lytic replication also promotes the progression of KS tumors [5]. There is currently no effective drug for eliminating latent KSHV infection and for treating KSHV-induced cancers. A comprehensive illustration of how the KSHV latent and lytic infections manipulate cellular metabolic pathways and metabolic sensors might help develop new treatments for KS tumors.

## KSHV reprograms glucose metabolism

**Fig. 1:**
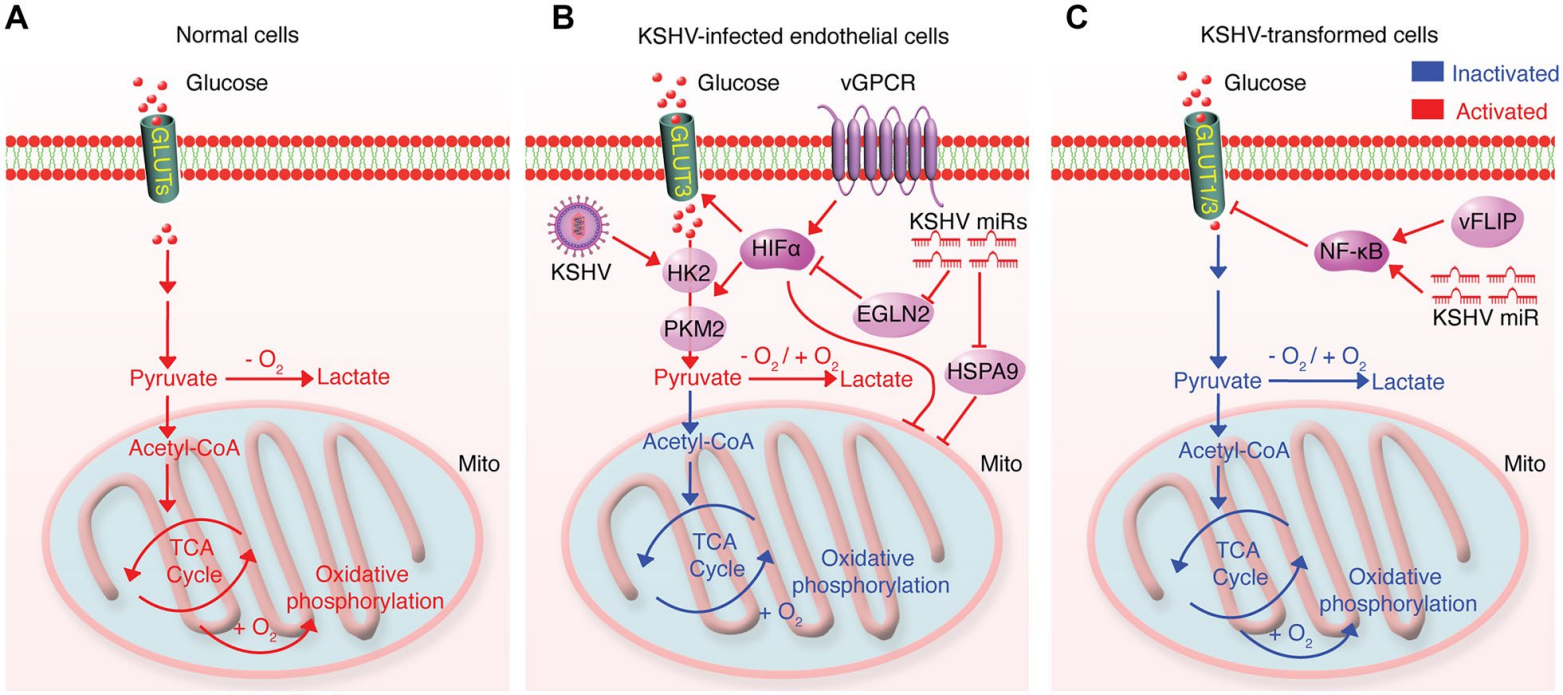
The glycolytic pathway in normal and KSHV-infected and -transformed cells. **A** The glycolysis is tightly linked to the oxygen level in normal cells. Under normoxia, the glucose is metabolized to pyruvate that fuels the TCA cycle and is coupled with oxidative phosphorylation to generate copious ATP. Under hypoxia, the glucose is metabolized to pyruvate, which is converted to lactate. **B** KSHV-encoded miRNA cluster and vGPCR induce the aerobic glycolysis in short-term KSHV-infected endothelial cells by upregulating HIF1α and inhibiting mitochondria. **C** KSHV-encoded miRNA cluster and vFLIP suppress the glycolysis and oxidative phosphorylation by NF-κB-mediated downregulation of GLUT1 and GLUT3

The first characterization of metabolic changes in tumors cells dated back to more than six decades ago when the Germany physiologist Otto Warburg observed that cancer cells consume a large amount of glucose and secrete excessive lactate even in the presence of oxygen yielding only 2 ATP per glucose [8]. In contrast, normal cells under normoxia preferentially catabolize glucose to pyruvate that is subsequently transported into mitochondria to fuel the tricarboxylic acid cycle (TCA) coupled with oxidative phosphorylation to generate 36 ATP [8] (Fig. 1A). Cancer patients undergoing aerobic glycolysis have poor survival [24]. In fact, positron emission tomography (PET)-based imaging for monitoring the uptake of a radiolabeled glucose analog, ^18^F-fluorodeoxyglucose (^18^F-FDG), has been successfully applied to diagnose and stage tumors. The combination of PET with ^18^F-FDG and computed tomography (^18^F-FDG PET/CT) provides valuable functional information regarding the uptake of glucose and glycolytic processes of cancer cells, which benefits cancer recurrence detection and treatments [25].

To examine the effect of KSHV infection on glucose catabolism, Delgado et al. infected telomerase-immortalized microvascular endothelial cells (TIME cells) and primary human dermal microvascular endothelial cells (hDMVECs) with KSHV for 48 h, and observed the induction of Warburg effect in KSHV-infected cells, which had increased glucose uptake and increased lactate secretion but decreased oxygen consumption in KSHV- versus mock-infected cells [26]. Inhibitors of aerobic glycolysis specifically induced apoptosis in KSHV-infected TIME cells, which was partially rescued by inhibiting oxidative phosphorylation, suggesting an essential role of Warburg effect for maintaining the survival of KSHV-infected cells. It is unclear if the carbon source is redirected to the TCA cycle and oxidative phosphorylation leading to cell death as a result of excess ROS production. Hexokinase 2 (HK2) is the rate-limiting enzyme that catalyzes the first step of glycolysis. HK2 was upregulated following 48 h of KSHV infection [26] but KSHV infection alone for 48 h failed to induce the glucose transporter 3 (GLUT3) that was only stabilized after adding hypoxia mimics. Interestingly, there was no notable difference in cell death between KSHV-negative Burkitt’s lymphoma BJAB versus KSHV-infected BJAB cells after inhibiting aerobic glycolysis [26], suggesting that there might be some cell type specificity in KSHV rewiring of the metabolic pathway. However, Singh et al. later showed that hypoxia-stabilized HIF1α was upregulated by KSHV-encoded vGPCR, a viral lytic gene, leading to increased aerobic glycolysis in KSHV-infected BJAB cells [27]. Additionally, the induction of hypoxia significantly changed the gene profiles of PEL cells involved in the metabolism of fatty acids and amino acids, suggesting that the KSHV-HIF1α axis might reprogram these metabolic pathways [27]. Similarly, Ma et al. reported that KSHV induced Warburg effect in human umbilical vein endothelial cells (HUVEC) by enhancing the HIF1α-mediated upregulation of pyruvate kinase 2 (PKM2), which is the key step in pyruvate production and aerobic glycolytic efflux [28]. Yogev et al. found that the KSHV-encoded miRNA cluster induced Warburg effect in lymphatic endothelial cells (LEC) by stabilizing HIF1α and inhibiting mitochondrial biogenesis through downregulating EGLN2 and HSPA9 [29] (Fig. 1B).

The above studies rely on the use of short-term KSHV infection systems or overexpression of KSHV-encoded miRNAs without taking into consideration of KSHV infection that doesn’t lead to cellular transformation. It is unclear how these systems might recapitulate the in vivo metabolic characteristics of KS tumors. In 2012 Jones et al. successfully infected, immortalized and transformed primary rat metanephric mesenchymal precursor cells (MM) with KSHV [30]. KSHV-transformed MM cells (KMM) are predominately latent and induce KS-like tumors in nude mice. This system makes it possible to delineate viral genes and cellular pathways required for KSHV-induced cellular transformation and tumorigenesis. Using this system, Zhu et al. found that KSHV-encoded miRNAs and vFLIP concomitantly activated the NF-κB signaling pathway to suppress aerobic glycolysis and oxidative phosphorylation by downregulating both GLUT1 and GLUT3 [31] (Fig. 1C). The independence on glucose is essential for KSHV-transformed KMM cells to survive in glucose-deprived stress tumor microenvironment. Importantly, downregulation of GLUT1 and GLUT3 were observed in the LANA-positive spindle tumor cells in human KS tumors, supporting the clinical relevance of the findings. Of note, the Warburg effect is likewise reduced in several KSHV-positive PEL cells and KSHV-infected BJAB cells [31], which might explain the lack of differential insensitivity of BJAB and KSHV-infected BJAB cells to aerobic glycolysis inhibition [26]. Clearly, the glycolytic pathway between KSHV-transformed and untransformed cells is different. However, it remains possible that these different observations could be due to the use of different cell types used in the studies or a reflection of different stages of KSHV infection. Although the alteration of glucose metabolism is sufficiently confirmed during KSHV infection, the exact mechanism of how the predominately expressed KSHV latent product(s) in KS tumors directly impacts glycolysis remains unclear.

## KSHV enhances glutaminolysis and the urea cycle efflux

**Fig. 2:**
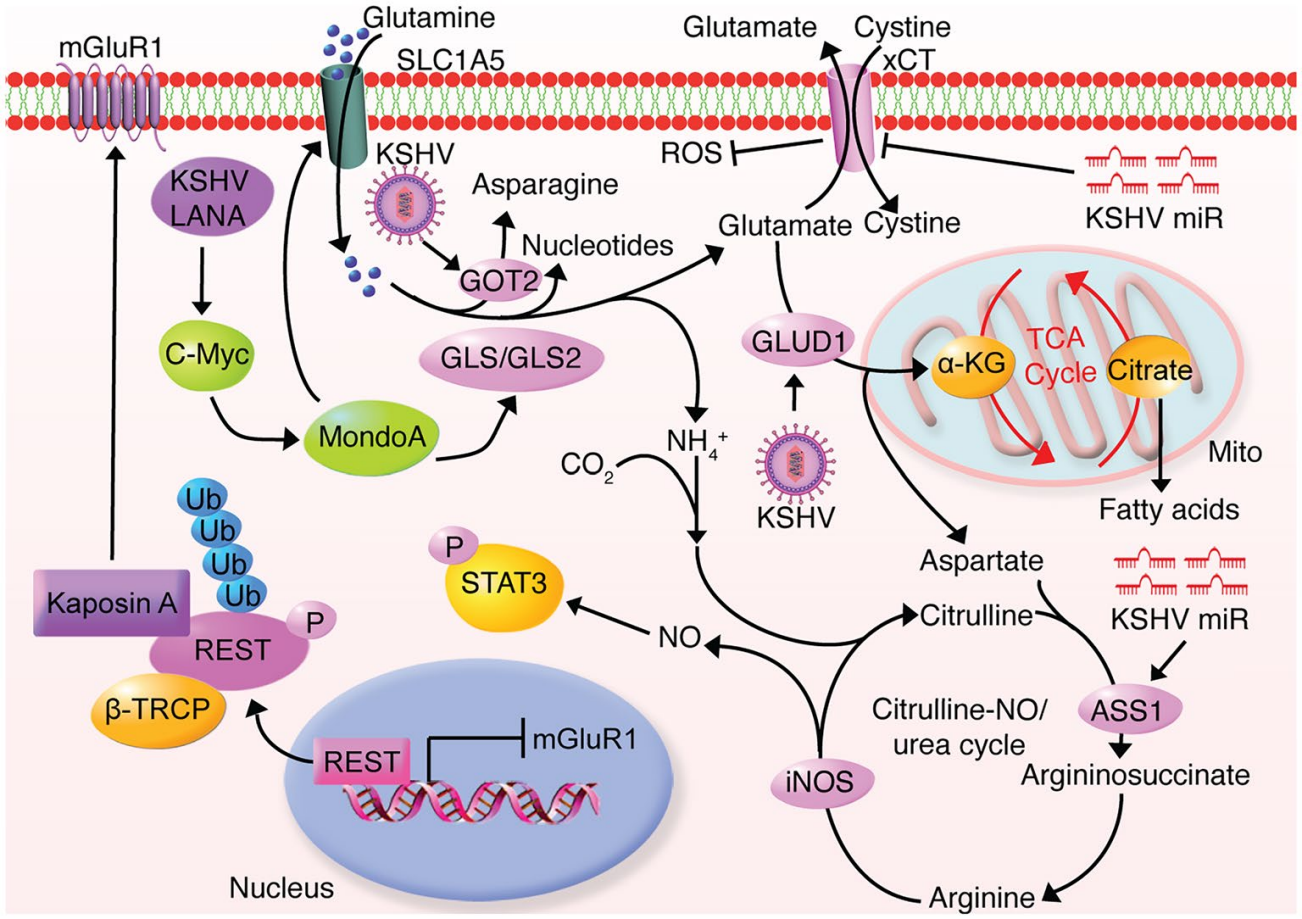
The glutaminolysis is upregulated in KSHV-infected and -transformed cells. KSHV promotes glutamine uptake by upregulating glutamine transporter SLC1A5; KSHV enhances glutaminolysis by upregulating several glutaminolytic enzymes including GLS1/2, GLUD1 and GOT2; KSHV Kaposin A upregulates mGluR1 to promote glutamate secretion while KSHV mediated upregulation of ASS1 via miRNAs and iNOS accelerates the citrulline-NO cycle leading to STAT3 activation

Glutamine is a very versatile amino acid, acting as energy fueling as well as a precursor for synthesizing many biological macromolecules. GLS and GLS2 hydrolyze glutamine to glutamate, which is exported by antiporter xCT coupled with cysteine import [32, 33]. Inhibition of GLS and GLS2 causes cell cycle arrest in several types of cancer cells, indicating the importance of glutaminolysis in tumorigenesis [34–36]. The promotion of glutaminolysis by xCT, which is upregulated by the KSHV miRNA cluster, replenishes intracellular glutathione and antagonizes reactive nitrogen species (RNS)-induced cell death [33, 37, 38] (Fig. 2). However, whether KSHV-mediated xCT upregulation promotes glutamine uptake, catabolism and glutamate secretion remains unknown.

Veettil et al. observed an increased secretion of glutamate into medium following KSHV latent infection, which was essential for the proliferation of KSHV-infected cells [39]. Mechanistically, RE-1 silencing transcription factor (REST), a transcriptional repressor of the metabotropic glutamate receptor 1 (mGluR1), interacts with and is sequestered by KSHV-encoded Kaposin A in the cytoplasm, accounting for mGluR1 upregulation in KSHV-infected cells [39]. However, more direct evidence is required to confirm the essentiality of mGluR1 in KSHV-induced glutamate secretion. Presumably, KSHV upregulation of xCT could also contribute to this process. Alternatively, the authors showed that KSHV LANA upregulated GLS by inducing c-Myc expression, which led to increased glutamine hydrolysis in KSHV-infected cells [39] (Fig. 2). Sanchez et al. further observed an increased uptake of glutamine following KSHV primary infection of TIME cells, which was mediated by KSHV-upregulated c-Myc that increased glutamine transporter SLC1A5 by transcriptionally inducing MondoA [40] (Fig. 2). Glutamine deprivation or SLC1A5 silencing selectively induced apoptosis of KSHV- rather than mock-infected cells, which was partially rescued by cell-permeable α-ketoglutarate (α-KG) anaplerosis [40]. As a key intermediate metabolite of glutaminolysis, α-KG is produced by two steps: GLS catalysis of glutamine to glutamate, which is then converted to α-KG through glutamate dehydrogenase 1 and 2 (GLUD1 and GLUD2). The rescue experiment demonstrated that KSHV-infected cells relied on glutaminolysis for survival as a result of glutamine fueling the TCA cycle.

Zhu et al. found that KSHV-transformed cells were addicted to glutamine but not glucose to sustain cell proliferation, survival and transformation [41]. Compared to primary cells, KSHV-transformed cells had increased consumption of glutamine and upregulation of several key glutaminolytic enzymes including GLS, GLUD1 and glutamic-oxaloacetic transaminase 2 (GOT2) [41] (Fig. 2). Intriguingly, whereas the supplementation of asparagine alone but neither any other NEAA nor α-KG fully rescued glutamine deprivation in the transformed cells while the combination of α-KG, glutamate and nucleosides mimicked the effect of asparagine [41] (Fig. 2). These results indicate that glutamine provides a nitrogen source for nucleotide synthesis and a carbon source for the TCA cycle and aspartate synthesis in KSHV-transformed cells [41]. The high consumption of glutamine in KSHV-transformed cells requires tight regulation and timely clearance of excess nitrogen to avoid accumulation of toxic byproducts, which can only be achieved by the citrulline-nitric oxide (NO) cycle. Indeed, KSHV-encoded miRNAs accelerate the citrulline-NO cycle by upregulating the rate-limiting enzyme argininosuccinate synthase 1 (ASS1) [42]. Knockdown of ASS1 suppressed cell proliferation and abolished colony formation in soft agar of KSHV-transformed cells, which was mimicked by inducible nitric oxide synthase (iNOS) knockdown [42]. Furthermore, ASS1 was required for KSHV activation of the STAT3 pathway by maintaining intracellular NO level, which was essential for KSHV-induced abnormal cell proliferation and transformation [42] (Fig. 2). Despite the advances in understanding the glutamine metabolism and the urea cycle in KSHV-infected and -transformed cells, the specific viral genes responsible for manipulating these pathways are elusive.

## KSHV promotes fatty acid synthesis

**Fig. 3:**
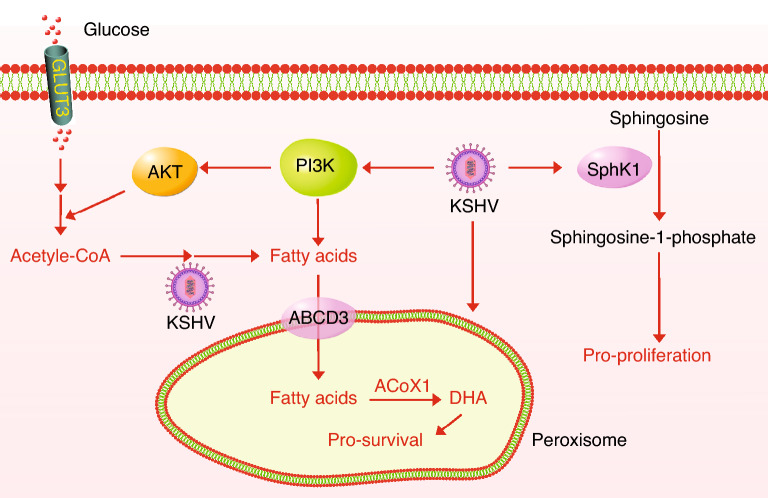
KSHV enhances lipogenesis and peroxisome-mediated ß-oxidation of lipids. The acetyl-CoA from KSHV-enhanced glycolysis leads to upregulated fatty acids synthesis; the ß-oxidation of lipids in peroxisomes is enhanced by short-term KSHV infection; and KSHV enhances the phosphorylation of sphingolipid sphingosine by upregulating the SphK1

Cancer cells have a high demand for fatty acids used for membrane synthesis. Acetyl-CoA is the obligate substrate for fatty acid synthesis (FAS), which is derived from the catabolism of glucose and glutamine. Bhatt et al. reported that KSHV-infected PEL cells upregulated fatty acid synthase and FAS compared to KSHV-negative primary B lymphocytes, which was essential for PEL cell proliferation and survival [43]. Additionally, the authors pointed out that the upregulated aerobic glycolysis in PEL cells was intimately linked to FAS as inhibition of one pathway blocked another. Both processes were highly dependent on the abnormally activated PI3K/AKT signaling pathway [43] (Fig. 3). One underpinning hypothesis for these observations is that aerobic glycolysis might provide the building blocks such as acetyl-CoA for FAS.

Sphingosine is a class of cell membrane lipids and can be phosphorylated by sphingosine kinases (SphK) to form a signaling lipid called sphingosine-1-phosphate (S1P) to elicit pro-proliferative and pro-survival signaling. Qin et al. showed that the inhibition of SphK1 specifically induced the apoptosis of PEL and KSHV-infected endothelial cells, hinting the important role of lipid metabolism [44]. Moreover, Angius et al. demonstrated that KSHV-infected HUVEC cells had increased the amount of neutral lipids, and the inhibition of cholesterol esterification decreased the tubular formation of KSHV-infected HUVEC cells, indicating that neutral lipids might be involved in neo-angiogenesis [45]. Nevertheless, there was no direct evidence linking KSHV infection to the altered lipogenesis until Delgado et al. profiled the global metabolites in cells before and after KSHV infection [46]. The authors showed at a molecular level that short-term KSHV infection of TIME cells induced nearly all intermediates for FAS and increased the de novo synthesis of long-chain fatty acids, which was essential for the survival of KSHV-infected cells [46] (Fig. 3). A follow-up study further confirmed the utilization and necessity of synthesized fatty acids in KSHV-infected cells by integrating transcriptomic, proteomic and metabolomic analyses [47]. It was found that cells infected by KSHV for 96 h had increased biogenesis of peroxisomes in which the β-oxidation and breakdown of fatty acids occurred [47]. The peroxisome-mediated lipid oxidation was essential for the survival of KSHV-infected cells as knockdown of two involved enzymes ABCD3 and ACOX1 specifically sensitized KSHV-infected cells to death [47] (Fig. 3). However, the functional importance of metabolites derived from peroxisome-mediated fatty acids oxidation and the mechanisms by which KSHV promotes FAS are unclear.

## The metabolic reprogramming during KSHV lytic replication

Considering the cellular environments that support KSHV latent and lytic replications are different, the metabolic activities during these two stages could differ. KSHV relies on cellular metabolites and energy for viral replication. Studies regarding the host metabolism during KSHV reactivation are limited. Sanchez et al. reported that glycolysis, glutaminolysis, and FAS are required for KSHV virion production and these metabolic pathways participate in distinct stages of viral life cycle [48]. Inhibitors of glycolysis and glutaminolysis specifically did not affect KSHV genome replication but altered KSHV early lytic gene expression at transcriptional and translational levels, respectively [48]. In contrast, FAS regulated the egress of KSHV virions without interfering with the genome replication [48]. Furthermore, FAS inhibition notably decreased the infectious KSHV virions in host cells, indicating that FAS might be critical for KSHV virion maturation and assembly [48]. These results indicate that there are different requirements for host metabolites during different stages of KSHV lytic replication. Nevertheless, these results obtained primarily through the use of inhibitors, often with limited specificity and efficiency, should be confirmed by genetic manipulation of critical metabolic enzymes. As efficient KSHV lytic replication is coupled with active DNA and RNA synthesis, a robust demand for nucleotide synthesis pathways during this phase of viral replication is expected.

## KSHV hijacks metabolic sensors

**Fig. 4:**
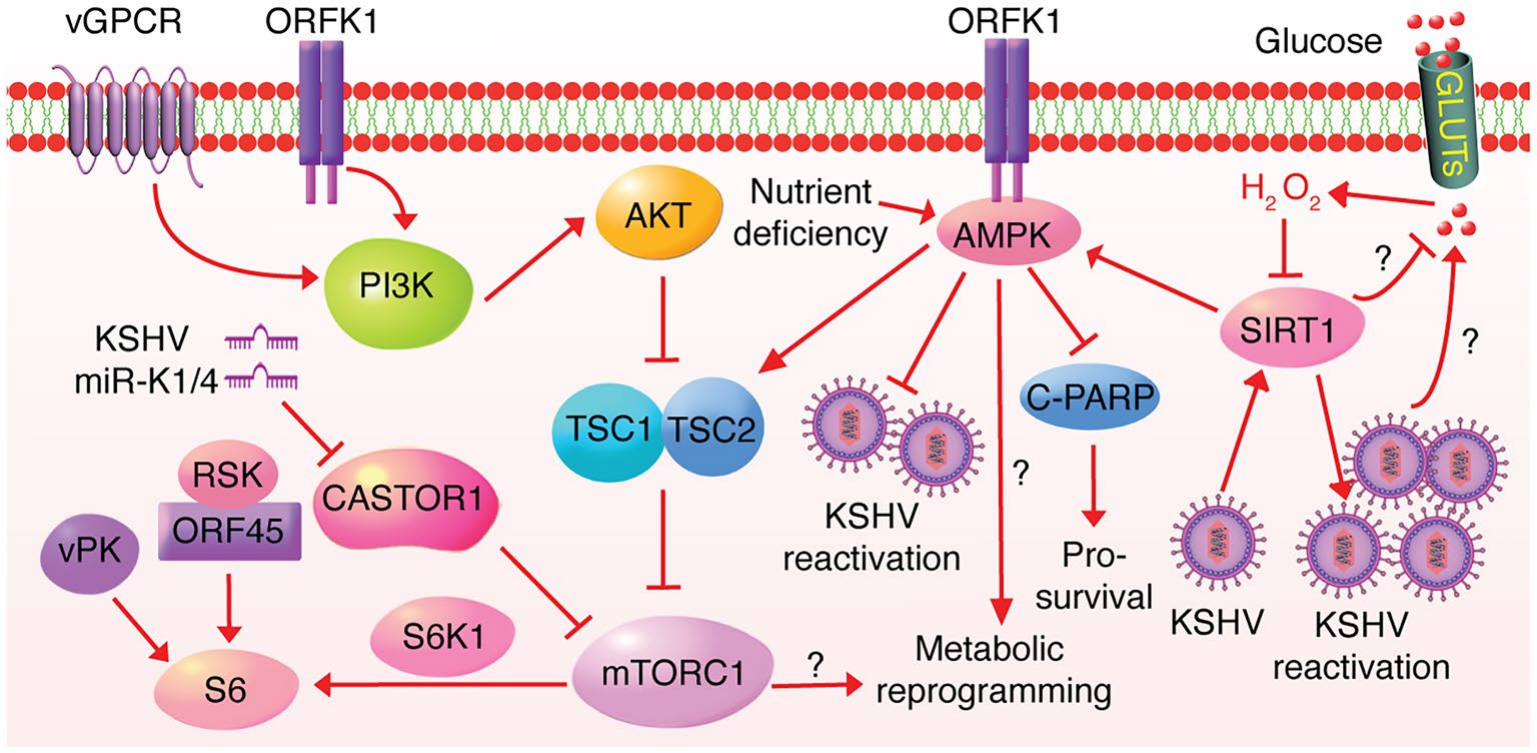
KSHV hijacks cellular metabolic sensors. KSHV-encoded vGPCR and glycoprotein K1 activate the mTORC1 signaling pathway through the PI3K-AKT cascade; KSHV vPK mimics the cellular S6K1 and directly phosphorylates S6, resulting in mTORC1 activation; KSHV miRNA-K1 and -K4 activate mTORC1 by downregulating CASTOR1; KSHV glycoprotein K1 directly interacts with and activates AMPK in nutrients-stressed condition; and KSHV activates AMPK by upregulating SIRT1

Although cancer cells are addicted to uptake of glucose and amino acids, they often encounter the nutrient scarcity because of the imbalance between increased consumption and limited supplies of nutrients. The aberrantly activated growth and survival signaling pathways play a major role in tumorigenesis at least by partially reprogramming the metabolism of cancer cells, allowing them to survive in nutrition-stressed conditions. Conversely, the reprogrammed metabolic pathways as well as the altered balance in metabolites in cancer cells also impact the metabolic sensors that sustain the uncontrolled proliferation of cancer cells. The most understood metabolite-sensing and signaling pathways are AMPK, sirtuins (SIRTs) and mTOR [49]. Many groups have reported that mTOR is highly activated and is essential for KSHV-induced tumorigenesis [50, 51]. Consistent with these observations, mTORC1 inhibitor rapamycin so far is the most effective therapy for KS tumors [52]. Several KSHV lytic genes including ORFK1, vPK (ORF36), ORF45 and vGPCR (ORF74) are reported to activate mTORC1 signaling pathway, whereas miR-K1 and -K4 are the only KSHV latent products reported so far that activate mTORC1 by directly downregulating the cytosolic arginine sensor for mTORC1 (CASTOR1) [53–57] (Fig. 4). KSHV miRNAs targeting CASTOR1 activates mTORC1 and relieves the tumor suppressive effect of CASTOR1 [58]. Inhibition of CASTOR1 function is also essential for breast cancer and possibly other types of cancer by AKT mediated RNF167-targeted degradation [59]. Normal cells use mTORC1 to sense multiple environmental inputs including oxygen, DNA damage, growth factors, energy and amino acids to maintain metabolic homeostasis [60, 61]. When nutrition is deficient, mTORC1 is inactivated to promote catabolism. Conversely, mTORC1 is activated and anabolism is enhanced if nutrients are surplus. Hence mTORC1 is largely involved in metabolic regulation, including glycolysis, protein synthesis, amino acids, nucleotide synthesis and lipogenesis [62]. As mTORC1 is constitutively activated in KS and PEL tumors, it is hypothesized that the metabolic pathways are likewise reprogrammed by the activated mTORC1 regardless of the extracellular nutrition status, although there is no direct evidence to prove it yet.

AMPK is another evolutionarily conserved metabolic sensor and is activated when energy supply is insufficient, resulting in an increase in the intracellular ratio of AMP/ATP or glucose shortage. Anabolism is inhibited by the activated AMPK leading to catabolism of glucose and lipids for ATP restoration. As a result, gluconeogenesis is inhibited, glucose uptake is increased and the mitochondrial biogenesis is prompted [63]. Elevated glycolysis coupled with oxidative phosphorylation restores the intracellular ATP level. Additionally, activated AMPK phosphorylates SREBP1c and ACC1/2 to inhibit lipids and cholesterols synthesis and simultaneously enhances lipids oxidation [64]. Of note, AMPK also directly and negatively regulates mTORC1 by activating TSC complex and inhibiting Raptor [65, 66], which greatly expands its role in controlling metabolism. Anders et al. showed that KSHV ORFK1 interacted with and increased AMPK activity under metabolic stressed conditions, which was critical for the survival and viral persistence of KSHV-infected cells [67] (Fig. 4). Additionally, Cheng et al. found that AMPK inhibition augments while AMPK activation restricts KSHV lytic replication during primary infection albeit AMPK activity was not significantly impacted [68]. It remains unclear how AMPK interferes with metabolic pathways to sustain the proliferation and survival of KSHV-infected cells.

NAD is a cofactor central to cellular metabolism and is composed of two forms: oxidized and reduced forms, abbreviated for NAD^+^ and NADH. Catabolism of one glucose requires two molecules of NAD^+^, producing two NADH in addition to two hydrogen ions and two molecules of water. The sirtuin family is composed of seven members from 1 to 7, which use NAD^+^ a co-substrate for enzymatic activities, are class III histone deacetylases (HDACs) functionally linked to cellular metabolism, and hence are regarded as metabolic sensors. Among them, SIRT1 is the most well-studied, and has a critical role in cancer because it inhibits glycolysis and stimulates fatty acids [69]. In PEL and KSHV-transformed cells, SIRT1 is significantly upregulated and positively regulates AMPK to sustain cell survival and resist c-PARP-induced apoptosis. The KSHV upregulation of SIRT1 is consistent with the inhibition of aerobic glycolysis observed in these cells [70, 71]. Additionally, SIRT1 epigenetically suppresses RTA such that the inhibition of SIRT1 reactivates KSHV in PEL cells [72]. Consistently, the high concentration of glucose forces the production of hydrogen peroxide (H_2_O_2_), leading to SIRT1 downregulation and hence KSHV reactivation [73] (Fig. 4). Hence, these metabolic sensors link KSHV life cycle to cellular metabolic state. Whether the activated SIRT1-AMPK signaling pathway partially arises from the suppressed aerobic glycolysis in KSHV-induced cancer cells and whether other SIRTs also mediate the survival of KSHV-infected cells and KSHV life cycle remain unknown.

The notion that KSHV hijacks the metabolic sensors including mTOR, AMPK, SIRT1 and CASTOR1 has been established. Nevertheless, whether KSHV hijacks these sensors to regulate host cell metabolism during latent and lytic infection remains unclear. Moreover, how KSHV-infected cells integrate the signals sensed by mTOR, AMPK, SIRT1 and CASTOR1 to sustain anabolic proliferation and survival is of great interest for understanding the mechanisms of viral persistence and KSHV-induced oncogenesis.

## Conclusion

Cancer cells sustain their uncontrolled proliferation in nutrients- and oxygen-deficient tumor microenvironments through reprogramming cellular metabolic pathways and sensors, which optimize their responses to environmental inputs and stress. While most studies concentrate on the glucose and glutamine metabolism, cancer cells usurp a great variety of other nutrients, for example, cysteines, vitamins, trace metals, and proline. The diversity of nutrients and the complexity of cellular responding circuits complicate our understanding how nutrients contribute to tumorigenesis. Viruses are absolute parasites that depend on the energy and macromolecules of host cells for their infection and spread. Oncogenic KSHV infection induces cellular transformation by altering host cell metabolism, which is essential for maintaining cell survival and viral persistence. However, the delineation of the underpinning mechanisms is instead confounded by the multiple stages of KSHV infection. A simple example is the differential glucose catabolism observed by two groups in short-term KSHV-infected endothelial and persistent KSHV-transformed cells, respectively. The application of ^18^F-FDG PET/CT technique to monitor the glycolytic efflux might reveal the true glucose metabolism in KS and PEL patients. However, it is also important to keep in mind that cancer cells and their microenvironment might have different metabolic demands, which could confound these assessments. Several KSHV-encoded products might be involved in reprogramming the glycolysis, glutaminolysis and FAS by activating specific cellular pathways. However, there is so far no experimental evidence to indicate the direct involvement of viral genes except miRNAs in regulating the host metabolic enzymes and remodeling the metabolic pathways. Most studies so far have only demonstrated the regulation of host cell signaling pathways by viral products and hence potential involvement in rewiring the cellular metabolic profiles [74]. Hence, how KSHV directly regulates host cellular metabolism remains largely unclear. Additionally, as nucleotides are indispensable for KSHV lytic replication and KSHV-induced cell proliferation and cellular transformation, studies demonstrate how the viral genes rewire the host cell nucleotides synthesis pathways are urgently required. A better understanding of the underlying mechanisms and a global screening to define other metabolic changes in KSHV-infected and -transformed cells are required, which could provide the scientific basis for developing new therapies against KS and PEL tumors. Despite substantial progresses, we are only at the beginning of understanding how KSHV reprogramming of host cell metabolic pathways contributes to tumorigenesis.

Numerous studies have investigated how KSHV evolves to usurp the host metabolic resources, funneling them towards reproducing virion progeny and transforming host cells. However, how cells utilize the metabolic countermeasures to sense and antagonize viral infections and how the host cell metabolic pathways impact KSHV infection and replication are less studied. It has been reported that high glucose could induce KSHV reactivation and KS is clinically correlated with diabetes mellitus [75–77]. It is reasonable to speculate that the rewired metabolic pathways might similarly affect viral gene expressions and life cycle. Additionally, whether KSHV dysregulation of metabolic sensors such as mTOR, AMPK, SIRT1 and CASTOR1 contribute to the metabolic alterations in host cells is unknown. A comprehensive mapping of the KSHV-dysregulated host cell metabolic networks should be attempted in order to fully exploit the potential of targeting these altered pathways for therapeutic intervention of KSHV-induced cancers.

## Data Availability

Not applicable.

## Abbreviations

KSHV: Kaposi’s sarcoma-associated herpesvirus
KS: Kaposi’s sarcoma
PEL: Primary effusion lymphoma
MCD: Multicentric Castleman’s diseases
KICS: KSHV-associated inflammatory cytokine syndrome
RTA: Replication and transcriptional activator
TCA: Tricarboxylic acid cycle
ROS: Reactive oxygen species
PET: Positron emission tomography
TIME cells: Telomerase-immortalized microvascular endothelial cells
hDMVECs: Primary human dermal microvascular endothelial cells
HK2: Hexokinase 2
GLUT3: Glucose transporter 3
HUVEC: Human umbilical vein endothelial cells
PKM2: Pyruvate kinase 2
LEC: Lymphatic endothelial cells
MM: Primary rat metanephric mesenchymal precursor cells
KMM: KSHV-transformed MM cells
RNS: Reactive nitrogen species
REST: RE-1 silencing transcription factor
mGluR1: Metabotropic glutamate receptor 1
α-KG: α-Ketoglutarate
GLUD1 and GLUD2: Glutamate dehydrogenase 1 and 2
GOT2: Glutamic-oxaloacetic transaminase 2
NO: Nitric oxide
ASS1: Argininosuccinate synthase 1
iNOS: Inducible nitric oxide synthase
FAS: Fatty acid synthesis
SphK: Sphingosine kinases
S1P: Sphingosine-1-phosphate
SIRTs: Sirtuins
CASTOR1: Cytosolic arginine sensor for mTORC1
HDACs: Histone deacetylases
H2O2: Hydrogen peroxide
